# Amplified insert assembly: an optimized approach to standard assembly of BioBrick^TM^ genetic circuits

**DOI:** 10.1186/1754-1611-5-17

**Published:** 2011-12-16

**Authors:** Michael A Speer, Tom L Richard

**Affiliations:** 1 Department of Agricultural and Biological Engineering, The Pennsylvania State University, 249 Agricultural Engineering Bldg., University Park, PA 16802, USA

**Keywords:** BioBrick, Synthetic biology, PCR, amplification, insert, vector, assembly, DpnI

## Abstract

A modified BioBrick™ assembly method was developed with higher fidelity than current protocols. The method utilizes a PCR reaction with a standard primer set to amplify the inserted part. Background colonies are reduced by a combination of dephosphorylation and digestion with DpnI restriction endonuclease to reduce vector and insert background respectively. The molar ratio of the insert to vector in the ligation was also optimized, with the accuracy of the transformed construct approaching 100%.

## Background

**Figure 1:**
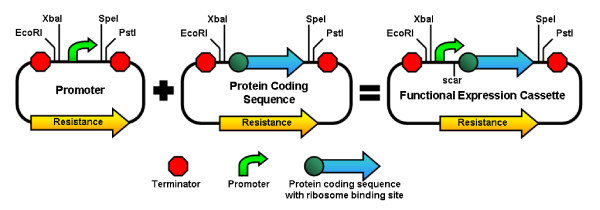
**Assembly of Parts Using the BioBrick Standard Format**. The promoter and the protein coding sequence, initially existing as parts on separate plasmids, are digested and ligated to form a composite part on one plasmid. Because XbaI (TCTAGA) and SpeI (ACTAGT) form compatible ends (CTAG), the two individual parts are connected by a mixed restriction site (ACTAGA) that is not recognized by any restriction endonuclease and is called the "scar". The individual parts may exist on plasmids with different resistances and the final composite parts may or may not be on a plasmid with the same resistance as its constituent parts.

The methods used for controlled modification of genetic material have experienced several major improvements since the initial development of recombinant DNA techniques in the early 1970s [1-4]. While there have been many efforts to simplify and standardize the genetic assembly process [5-7], one format that has gained widespread acceptance is the BioBrick™[8]. BioBrick assembly is commonly applied within a synthetic biology conceptual framework that abstracts and classifies basic functional units of genetic material (promoters, ribosome binding sites, protein coding sequences like reporters, terminators, etc.) as "parts" that can be assembled into "devices" that create new functionality, and higher level "systems" to accomplish complex tasks [9]. While the BioBrick standard has been revised several times [10-12], the basic format has remained the same. Each "BioBrick part" is contained on a plasmid and is flanked by four unique restriction sites, (two 5' and two 3') with the inner restriction sites that can be cut by two endonucleases yielding compatible ends. Using this format, individual parts may be selectively digested and then ligated together to form new composite parts or devices while preserving the format, connecting the individual parts by a benign mixed restriction site known as a "scar" (Figure 1). The chief utility of this technology is that all parts using the same format are interchangeable and composite parts can be recombined in the same way as individual parts [8]. This has allowed entire catalogs of compatible biological parts to be developed [9,13]. The major limitation of this format is that composite parts must be assembled piece by piece. To make this system useful and affordable, easy and reliable standard methods of performing these assemblies have been developed [14].

**Figure 2:**
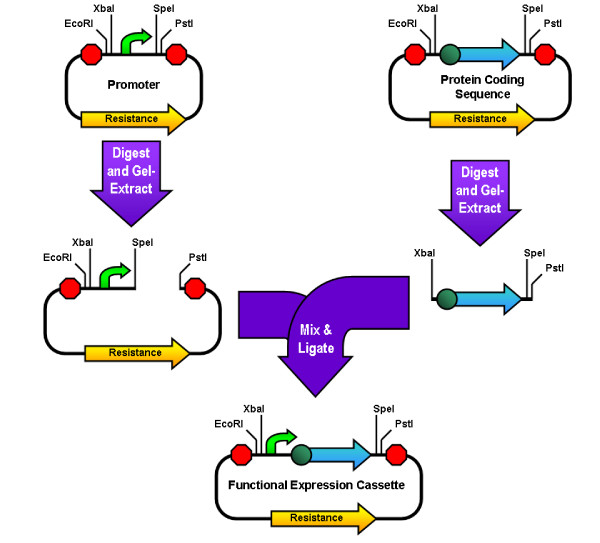
**Standard Assembly**. This figure illustrates a typical standard assembly. The plasmid containing the promoter is digested with SpeI and PstI, and the plasmid containing the coding sequence is digested with XbaI and PstI. Both of these digests are separated using agarose gel electrophoresis and the relevant parts are recovered from the gel. The gel extracts are then ligated together and the circular product is transformed into cells. Because of the necessity for gel electrophoresis, performing this same assembly using the small-sized promoter as the insert instead of the protein coding sequence would be extremely difficult.

The original method used to perform "bio-bricking" is called the standard assembly method. In using this method, one part is designated the insert and is digested out of its plasmid using enzymes EcoRI and SpeI or XbaI and PstI [8]. The other part remains in its plasmid, but the plasmid is opened up using compatible enzymes: EcoRI and XbaI or SpeI and PstI respectively (Figure 2). Upon successful double ligation the inner XbaI and SpeI sticky ends will form a benign scar and the two parts will be adjacent to one another in the final circular construct. This protocol requires the following steps: 1) extraction of plasmids containing the two parts to be assembled; 2) digestion of the plasmids to create compatible ends on DNA fragments; 3) separation of the digested DNA by agarose gel electrophoresis; 4) extraction of the selected fragments from the gel; 5) ligation of the fragments together; and 6) transformation of the ligated plasmid product into cells. This process has many advantages: the parts can all be on plasmids with the same antibiotic resistance; thermostable enzymes (e.g. BglII and BamHI) can be used; and only two successful ligations are necessary to achieve a circularized plasmid. However, there is a key disadvantage: the gel electrophoresis and extraction are difficult to automate and result in poor yields of purified DNA, especially for smaller parts.

**Figure 3:**
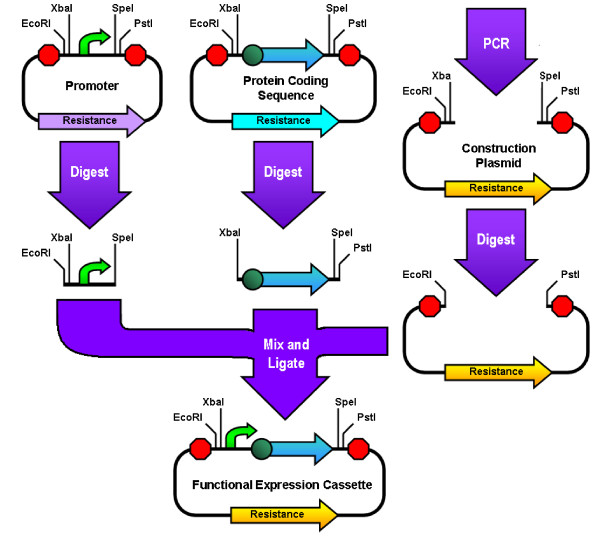
**Three Antibiotic (3A) Assembly**. Using this method of assembly, the promoter is digested with EcoRI and SpeI and the coding sequence is digested with XbaI and PstI. A PCR linearized "construction plasmid" with a different antibiotic resistance than the others is also digested with EcoRI and PstI and may or may not be treated with DpnI and phosphatase to remove some of the background plasmids. These three digests are then ligated together and the circular product of that ligation is transformed into cells.

To address many of these problems, the three antibiotic (3A) assembly method was developed [15]. This method successfully eliminates the necessity for gel electrophoresis and the associated costs/problems/time and enables the automated assembly of small parts. Using this method the two plasmids containing the parts to be assembled are extracted as before, but both parts (designated "prefix" and "suffix") are digested out of their respective plasmids and then inserted into a third plasmid in a three way ligation. This third plasmid is called the "construction plasmid" and has a different antibiotic resistance than the part plasmids (Figure 3). Various means are employed to eliminate background transformation of the construction plasmid including the insertion of a specific "cell death" gene [16-18] and the use of PCR-linearized plasmid backbone [14]. While this 3A Assembly greatly simplifies the assembly of biological parts, it has some disadvantages as well. The three-way ligations are less efficient and produce comparatively fewer circularized products and fewer transformants. It is also possible for these backbones to be ligated to the construction plasmid in place of the desired part because the digested prefix and suffix plasmid backbones are included in the ligation. Both of these problems lead to a loss of accuracy and the need to screen and sequence a number of colonies.

We proposed and critically tested an alternate BioBrick assembly method called Amplified Insert Assembly to eliminate many of these problems. This method combines the functional simplicity of the standard assembly method with the ease and flexibility of 3A Assembly and is compatible with any BioBrick standard in which the restriction endonucleases are able to be heat inactivated. It is based on the original standard assembly method in that it uses a double ligation to insert one part, designated the "insert", adjacent to another part that remains in its plasmid, and thus is the designated "vector". The need for gel electrophoresis is eliminated because the insert is amplified from its original plasmid using high-fidelity PCR, and background transformation is eliminated by simple enzymatic treatments. Very small parts can also be assembled with ease because this PCR step adds sufficient length to allow purification using a DNA binding column. While these combined treatments eliminate the need for gel electrophoresis and enable the assembly of small parts, they also substantially decrease the required starting quantity of DNA, generally making use of the BioBrick repository more affordable and versatile.

Several aspects of the amplified insert (AI) assembly protocol will be familiar to those using standard cloning techniques, as they are very similar to the processes involved in typical cloning of DNA [19]. This new assembly protocol is differentiated by 1) the use of common flanking primers which preserve, during amplification, the existing restriction sites flanking the BioBrick part, 2) these primers also add sufficient length of DNA (~300 bp) to the amplified part to allow even very small parts (e.g. RBSs) to be purified easily using a DNA binding column, 3) clean-up of the amplified insert by DpnI digestion, and 4) the elimination of background vector by the dephosphorylation of the vector DNA.

## Results

**Figure 4:**
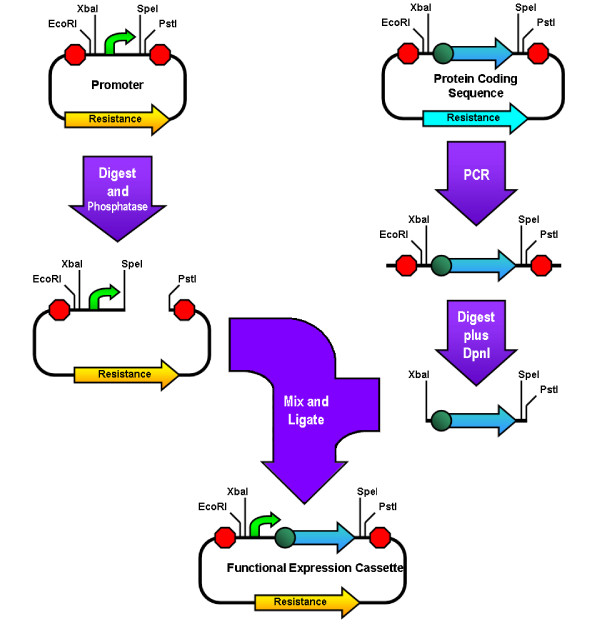
**Amplified insert Assembly**. The insert part is amplified from its plasmid using high-fidelity PCR and the purified PCR product is digested with XbaI, PstI, and DpnI. Meanwhile the vector part's plasmid is digested with SpeI and PstI and phosphatased. The products of these two digestions are then ligated and the circular product is transformed into cells. This assembly can easily be done in reverse manner using the promoter as the insert and leaving the coding sequence in its vector, thus providing additional flexibility.

The protocol for utilizing the AI assembly method is simple and fast. First, the plasmid DNA for both parts is purified from an overnight culture. The insert DNA is then amplified from its plasmid using 25 cycles of high-fidelity PCR. This amplification uses standard primers that anneal to all BioBrick plasmids and eliminate the need to order custom oligos for each assembly, as required by other PCR based assembly methods [20-22]. While this PCR is running, the vector plasmid is digested with either EcoRI and XbaI or SpeI and PstI. After two hours of digestion, the vector is also treated with Antarctic Phosphatase to remove the terminal phosphates in the cut vector and prevent self-ligation. When the PCR product is finished it is purified using a DNA binding column and then digested for one hour with either EcoRI and SpeI or XbaI and PstI to complement the vector. DpnI is also added to this digest as a third restriction endonuclease. Once all digests are heated to inactivate the endonucleases (80°C for 20 min), they are ligated using T4 Ligase at a molar ratio of 4:1 (insert:vector), transformed into competent cells, and plated onto media of appropriate resistance. This method is illustrated in Figure 4.

**Figure 5:**
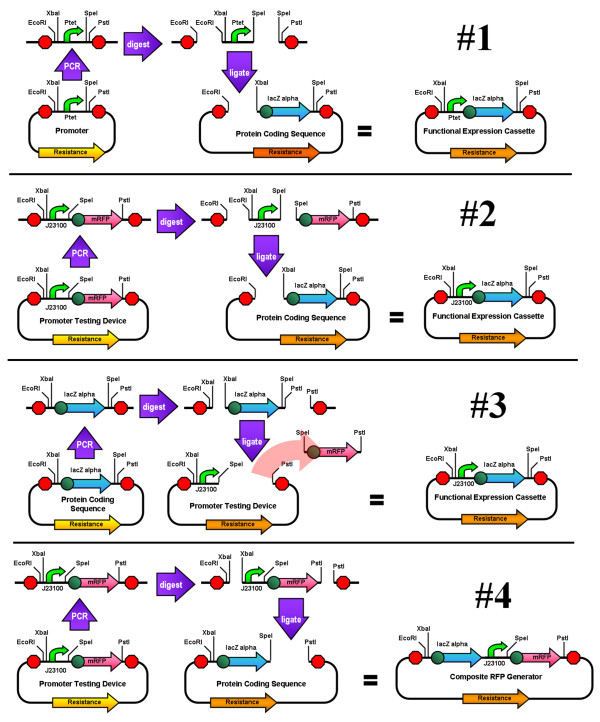
**Experimental Amplified Insert Assemblies**. 1) The Ptet promoter is amplified from its plasmid and inserted 5' of *lacZ α *with RBS. The amplified fragment is 350 bp and the insert size is 50 bp. 2) The J23100 constitutive promoter is amplified from its testing device and inserted 5' of *lacZ α*. The amplified fragment is 1200 bp and the insert size is 40 bp. 3) The *lacZ α *is amplified from its plasmid and is inserted 3' of the J23100 promoter while replacing the existing coding sequence in the testing device and restoring the standard BioBrick format. The amplified fragment is 830 bp and the insert size is 530 bp. 4) The entire promoter testing device is amplified from its plasmid and inserted 3' of *lacZ α *creating a composite mRFP generator and testing device. The amplified fragment is 1200 bp and the insert size is 900 bp.

The high accuracy of this assembly is enabled by the use of DpnI restriction endonuclease to eliminate insert background and Antarctic Phosphatase to eliminate vector background. This additional processing is an enzymatic substitute for the mechanical separation of gel electrophoresis, and can be performed concurrently to the restriction digest without requiring any additional time. DpnI restriction endonuclease is a frequent blunt cutter (recognition site GATC) but only cuts sites with a methylated adenine residue [23]. Many cloning strains of *E. coli *are positive for Dam methyltransferase which specifically methylates this GATC site [24], making it susceptible to DpnI cleavage. Because the synthetic DNA created during PCR amplification is not methylated, the DpnI only cuts the template DNA. Thus the PCR followed by digestion with DpnI provides an enzymatic amplification and clean-up of the BioBrick insert. Similarly, the use of a phosphatase prohibits the cut vector DNA from re-ligating by removing the terminal phosphate groups necessary to bond DNA bases. To verify and quantify the accuracy and efficiency of AI assembly, promoters and protein coding sequences of various sizes were inserted to create functional BioBrick expression cassettes for the *lacZ α *fragment [25] and *mRFP*[26], allowing the constructs to be screened by color. In addition to the traditional additive assembly procedure, AI assembly was also used for coding sequence replacement, which is commonly used for promoters that are contained in testing devices (Figure 5). The molar ratios of insert to vector were adjusted to determine the optimum for this protocol, as this ratio has been shown to have a substantial effect on ligation success [27]. As a comparison, three antibiotic (3A) assembly was also performed as instructed in the BioBrick™ assembly kit (Ginkgo BioWorks, Boston MA, USA). For all assemblies the insert parts were initially contained on ampicillin resistant BioBrick plasmids, and all composite parts were contained on chloramphenicol resistant plasmids. This allowed the separate assessment of insert background in AI assembly by plating the transformation product on ampicillin plates. This background number allows us to quantify the additional number of incorrect colonies if the vector and insert plasmids both had the same resistance. This background assessment was not done for 3A assembly, as this method requires that the construction plasmid have a different resistance than the prefix or suffix and therefore the insert background is theoretically insignificant as it would not appear on plates of the correct resistance.

The results of these tests are summarized in Table 1. Non-colored chloramphenicol resistant colonies are enumerated as "incorrect" and colonies that appear on ampicillin plates are enumerated as "background". The 3A assemblies were each transformed twice (column purified/concentrated and unpurified) to ensure sufficient colonies for enumeration, as the unpurified 3A ligation produced only a few colonies. The unpurified transformation data are given first. All AI assembly ligations were transformed unpurified. For the AI assembly tests, various combinations of placing the insert 5' (ligations #1 and #2) or 3' (ligations #3 and #4) of the vector part are represented. Sequences of the assembled parts are available in [Additional File 1].

**Table 1:** Transformation Results

Amplified Insert Assembly								
**#**	**Ligation Inputs**			**Results**				
	
	**Insert**	**Vector**	**Ratio**	**Color**	**Correct**	**Incorrect**	**Background**	**% Accuracy**

	Ptet	*lacZ α*	1:1	Blue	819	6	1	99.6 ± 0.9%
	
1	Ptet	*lacZ α*	2:1	Blue	868	13	1	98.4 ± 0.7%
	
	Ptet	*lacZ α*	4:1	Blue	793	0	1	**99.9 ± 0.2%**

	PJ23100	*lacZ α*	1:1	Blue	778	3	3	99.2 ± 0.6%
	
2	PJ23100	*lacZ α*	2:1	Blue	1454	5	1	99.6 ± 0.8%
	
	PJ23100	*lacZ α*	4:1	Blue	607	0	1	**99.8 ± 0.3%**

	*lacZ α*	PJ23100	1:1	Blue	391	12	6	95.6 ± 2.2%
	
3	*lacZ α*	PJ23100	2:1	Blue	547	2	3	**99.1 ± 0.7%**
	
	*lacZ α*	PJ23100	4:1	Blue	465	5	1	98.7 ± 3.3%

	mRFP Driver	*lacZ α*	1:1	Red	676	34	5	94.6 ± 3.4%
	
4	mRFP Driver	*lacZ α*	2:1	Red	1329	24	1	98.2 ± 0.9%
	
	mRFP Driver	*lacZ α*	4:1	Red	1761	22	6	**98.4 ± 1.0%**

**Three-Antibiotic Assembly**								

**#**	**Ligation Inputs**			**Results**				
	
	**Prefix**	**Suffix**	**Vector**	**Color**	**Correct**		**Incorrect**	**% Accuracy**

5	PJ23100	*lacZ α*	pSB1C3	Blue	8		1	89%

5*	PJ23100	*lacZ α*	pSB1C3	Blue	418		43	**90.7 ± 0.6%**

6	*lacZ α*	mRFP Driver	pSB1C3	Red	3		1	75%

6*	*lacZ α*	mRFP Driver	pSB1C3	Red	174		29	**85.7 ± 4.6%**

The cumulative number of colonies after performing assemblies in triplicate on three separate days are shown. 3A ligations marked with an asterisk (*) were purified and concentrated before transformation. The highest % accuracy is bolded for each ligation reaction.

## Conclusions

Based on these results shown in Table 1 it can be seen that the amplified insert assembly method produces the desired composite part with greater accuracy when compared to 3A assembly. There is also a greater trend toward success at the higher molar ratios, with the 4:1 (insert:vector) ratio giving greater accuracy in three out of the four assemblies tested. Furthermore, it can be seen that the amplified insert assembly is equally adept at performing the standard additive assembly (ligations #1 and #4) as well as coding sequence replacement (ligations #2 and #3). These results also show a trend toward decreased accuracy when the insert is placed behind the vector part (ligations #3 and #4). This could be due to the lower activity of PstI in the reaction buffer used (75% compared to 100% for all other enzymes according the NEB's buffer chart). While this can be slightly frustrating, it also illuminates the fact that the amplified insert assembly method allows an added level of choice in determining which enzymes to use. In contrast, with 3A assembly all enzymes must be used for every assembly. While it should be noted that the extremely low numbers of colonies given by the unpurified 3A ligation is a substantial limitation of this method, this limitation is readily eliminated by a quick purification step. Similar assemblies were also performed using the standard assembly method with gel electrophoresis. These assemblies had an accuracy of 94%, but only yielded an average of three colonies per plate, and none of the assemblies involving small inserts yielded any colonies.

These results show that the amplified insert assembly method preserves the functional simplicity (and therefore accuracy) of the original standard assembly method while possessing several positive characteristics of 3A assembly, including the elimination of gel electrophoresis and the ability to assemble small parts. While the PCR step can sometimes add up to two hours to the total assembly time, the total time from cultures to plating is usually under six hours, and the hands-on time is only slightly increased when compared to 3A assembly. Furthermore, because this protocol allows the amplification of insert from a small amount of DNA, plasmid preps of common parts (e.g. promoters, terminators, and RBSs) can be kept as a stock and used repeatedly as insert template. If the PCR is performed in advance from a plasmid stock, the total time required for AI assembly is four hours. A comparison of the various BioBricking methods can be found in Table 2.

**Table 2:** Comparison of Alternative Assembly Methods

	Standard Assembly	Three Antibiotic Assembly	Amplified Insert Assembly
Plasmid Extraction	X	X	X

Restriction Digest	X	X	X

Phosphatase Treatment		X	X

DpnI Digest			X

Gel Electrophoresis	X		

PCR		X	X

Ligation	X	X	X

Purification	After Gel	Before Transformation	Before Digestion

Accuracy	Depends on size: Gel extraction difficult for small inserts	89%	99%

Because this procedure uses PCR to amplify the insert, there is an inherent risk of causing mutations in the part sequence. To mitigate this risk a few steps should always be taken: 1) a high fidelity polymerase should always be used; 2) the number of PCR cycles should be kept at or below 30; and 3) the final construct should always be sequenced to detect any mutations. It also occasionally happens that PCR can unexpectedly fail for a number of reasons, but because our reaction uses the same polymerase and primers for each assembly, the use of a standard 2× stock can drastically reduce the user error involved in the amplification process. Despite these risks we have yet to observe a mutated plasmid in over 100 successful sequenced assemblies, and have found this method to be a quick, effective, and reliable way to perform all types of BioBrick assembly.

## Methods

For all experiments *E. coli *strain DH5α was grown in SOB liquid medium [28] supplemented with appropriate antibiotic (100 μg/ml Ampicillin; 50 μg/ml Kanamycin; 35 μg/ml Chloramphenicol) or on 2% agar plates at 37°C supplemented with X-gal (200 mg/L) and the appropriate antibiotic. All parts and plasmids used in this experiment were obtained from the Registry of Standard Biological Parts http://partsregistry.org/Main_Page, and a list of the parts and plasmids used is provided in Table 3. Plasmids were extracted using the E.Z.N.A Plasmid Mini Kit (Omega Bio-Tek, Norcross, GA, USA). DNA was quantified using a NanoDrop 2000 spectrophotometer (Thermo Scientific, Wilmington, DE, USA).

**Table 3:** Parts and Plasmids

Part#	Description	Size (bp)	Use	Plasmid	Resistance
BBa_I732018	lacZ α with a strong ribosome binding site	534	Insert	pSB1AK3	Ampicillin, Kanamycin

BBa_J23100	Strong constitutive promoter in mRFP testing device	35 + 890	Insert	pBca1020	Ampicillin

BBa_R0040	TetR repressible promoter	54	Insert	pSB1A2	Ampicillin

BBa_I732018	lacZ α with a strong ribosome binding site	534	Vector	pSB1C3	Chloramphenicol

BBa_J23100	Strong constitutive promoter in mRFP testing device	35 + 890	Vector	pSB1C3	Chloramphenicol

The plasmids used are described according to the parts registry number www.partsregistry.org and the size of the BioBrick part contained on the plasmid. Parts given two sizes are promoters contained on promoter testing devices that have an mRFP coding sequence between the SpeI and PstI sites. The sizes are given as the promoter and mRFP gene respectively.

### Amplified insert assembly

#### PCR

Parts were amplified via PCR using the high fidelity Vent^® ^DNA Polymerase (New England BioLabs, Ipswich, MA, USA) and run according to the suppliers protocol (2011) in 1× Thermopol Buffer (2 mM Mg^2+^) for 25 cycles (25 sec@94°C, 25 sec@58°C, extension (1 min/kb)@72°C). Primers VF2 (tgccacctgacgtctaagaa) and VR (attaccgcctttgagtgagc) were used that flank the restriction sites by approximately 125 bp and have a T_m _of 60°C. PCR cleanups were performed using the E.Z.N.A. Cycle-Pure Kit (Omega Bio-Tek, Norcross, GA, USA).

#### Digestion

All digests were performed in 50 μl volumes containing 1x NEBuffer 2 (New England BioLabs, Ipswich, MA, USA) and either 0.5 pmol of purified PCR product or 0.25 pmol of purified plasmid. Twenty units of each enzyme were used for the digests, except for SpeI and Antarctic Phosphatase which were used in 10 unit and 5 unit quantities respectively. Digests were performed according to the following scheme:

*To place the insert part in 5' of the vector part*. The vector was digested with EcoRI and XbaI for two hours (while the insert PCR was taking place) and then treated with Antarctic Phosphatase (supplemented with the specified buffer) for one hour while the purified insert was digested with DpnI, EcoRI, and SpeI;

*To place the insert 3'of the vector part*. The vector was digested with SpeI and PstI for two hours (while the insert PCR was taking place) and then treated with Antarctic Phosphatase (supplemented with the specified buffer) for one hour while the insert was digested with DpnI, XbaI, and PstI.

All digests were heat inactivated for 20 min at 80°C. All enzymes were purchased from New England BioLabs (Ipswich, MA, USA).

#### Ligation

Ligations were performed in 20 μl volumes using T4 Ligase (New England BioLabs, Ipswich, MA, USA) according to the manufacturers protocol (2011) for sticky ends. A total of 6 μl of unpurified digests was used for each ligation, with the molar ratios varied from 1:1 to 4:1 (insert:vector).

### Three-antibiotic (3A) assembly

#### vector construction

The backbone of the plasmid pSB1C3 was amplified and linearized via PCR using Phusion^® ^High-Fidelity DNA Polymerase (New England BioLabs, Ipswich, MA, USA). Phusion^® ^was used here because it has high processivity (4 kb/minute), a higher fidelity than Vent^®^, and works well for large parts or, in this case, a 2.5 kb plasmid backbone. However, because of its high processivity Phusion^® ^is not ideal for the amplification of small parts, which is why Vent^® ^was used elsewhere. Primers which bind in the BioBrick sites and have a T_m _of 61°C were used for 35 cycles according to the manufacturer's (2011) specifications. Constructs were purified using the E.Z.N.A. Cycle-Pure Kit and used immediately.

#### Digestion

0.25 pmol of DNA were used for all 50 μl digests. The prefix parts were digested with EcoRI and SpeI and the suffix parts were digested with XbaI and PstI. The linear construct plasmid was digested with EcoRI and PstI and also supplemented with DpnI and Antarctic Phosphatase to eliminate background as described above. During the phosphatase step, vector digests were supplemented with 6 μl of Antarctic Phosphatase buffer. All reactions were heat inactivated for 20 min at 80°C after three hours of digestion.

#### Ligation

2 μl of each digest (6 μl total) was added to the ligation mix as described above. The ligations were done in duplicate with one ligation being transformed in its unpurified state, while the other ligation was purified using an E.Z.N.A MicroElute Cycle-Pure Kit (Omega Bio-Tek, Norcross, GA, USA) and concentrated in a volume of 5 μl, which was used in its entirety to transform cells.

### Transformation and selection

5 μl of ligation product was added to 100 μl DH5α competent cells (1 × 10^7 ^cfu/μg DNA) and transformed using an Eppendorf 2510 electroporator (Hamburg, Germany) at 15,000 V/cm. Transformed cells were diluted into 900 μl prewarmed SOC medium [28] and let recover for 30-60 min at 37°C. 100 μl of this recovery solution was then plated in triplicate on chloramphenicol plates and singly on an ampicillin plate to assess background insert plasmid (for AI assembly only). The plates were developed for two days before color counts were made. Strains containing only *lacZα *(without a promoter), as well as the mRFP driver were also tested to ensure that they did not produce a false positive for the X-gal screen.

## List of abbreviations used

RBS: ribosome binding site; ORF: open reading frame; mRFP: monomeric red fluorescent protein; PCR: polymerase chain reaction; SOB super optimal broth; bp: base pairs.

## Competing interests

The authors declare that they have no competing interests.

## Authors' contributions

MAS conceived of and developed the idea of using amplified insert assembly for standard assembly of BioBrick parts. MAS and TLR designed the experiments and drafted the manuscript. Both authors read and approved the final manuscript.

## Authors' information

MAS is a Ph.D. candidate, graduate research assistant, and mentor for the Penn State iGEM team. TLR is a professor of agricultural and biological engineering and director of the Penn State Institutes of Energy and the Environment, a past-president and Fellow of the Institute of Biological Engineering, and also a mentor of the Penn State iGEM team.

## Supplementary Material

Additional file 1 **Gene Sequences**. This file contains the gene sequences of the parts used as well as the final constructs created by assembly. Click here for file
