# Supplementary material for: Amplified insert assembly: an optimized approach to standard assembly of BioBrickTM genetic circuits
Source: J Biol Eng. 2011 Dec 16;5:17. doi: 10.1186/1754-1611-5-17 (PMC3287150; doi:10.1186/1754-1611-5-17)
Supplement: Additional file 1 — Gene Sequences. This file contains the gene sequences of the parts used as well as the final constructs created by assembly. [file 1754-1611-5-17-S1.DOCX]

**Sequences of assembled parts are provided below.**

**BBa_I732018 *lacZα* with 5’ RBS**

aaagaggagaaatactagatgaccatgattacgccaagcgcgcaattaaccctcactaaagggaacaaaagctggagctccaccgcggtggcggcagcactagagctagtggatcccccgggctgtagaaattcgatatcaagcttatcgataccgtcgacctcgagggggggcccggtacccaattcgccctatagtgagtcgtattacgcgcgctcactggccgtcgttttacaacgtcgtgactgggaaaaccctggcgttacccaacttaatcgccttgcagcacatccccctttcgccagctggcgtaatagcgaagaggcccgcaccgatcgcccttcccaacagttgcgcagcctgaattaataa

**BBa J23100 Promoter in testing device**

ttgacggctagctcagtcctaggtacagtgctagctactagtgaaagaggagaaatactagatggcttcctccgaagacgttatcaaagagttcatgcgtttcaaagttcgtatggaaggttccgttaacggtcacgagttcgaaatcgaaggtgaaggtgaaggtcgtccgtacgaaggtacccagaccgctaaactgaaagttaccaaaggtggtccgctgccgttcgcttgggacatcctgtccccgcagttccagtacggttccaaagcttacgttaaacacccggctgacatcccggactacctgaaactgtccttcccggaaggtttcaaatgggaacgtgttatgaacttcgaagacggtggtgttgttaccgttacccaggactcctccctgcaagacggtgagttcatctacaaagttaaactgcgtggtaccaacttcccgtccgacggtccggttatgcagaaaaaaaccatgggttgggaagcttccaccgaacgtatgtacccggaagacggtgctctgaaaggtgaaatcaaaatgcgtctgaaactgaaagacggtggtcactacgacgctgaagttaaaaccacctacatggctaaaaaaccggttcagctgccgggtgcttacaaaaccgacatcaaactggacatcacctcccacaacgaagactacaccatcgttgaacagtacgaacgtgctgaaggtcgtcactccaccggtgcttaataacgctgatagtgctagtgtagatcgctactagagccaggcatcaaataaaacgaaaggctcagtcgaaagactgggcctttcgttttatctgttgtttgtcggtgaacgctctctactagagtcacactggctcaccttcgggtgggcctttctgcgtttata

**BBa_R0040 Tet Promoter**

tccctatcagtgatagagattgacatccctatcagtgatagagatactgagcac

**Ligation #1 Ptet driving LacZα expression**

tccctatcagtgatagagattgacatccctatcagtgatagagatactgagcactactagagaaagaggagaaatactagatgaccatgattacgccaagcgcgcaattaaccctcactaaagggaacaaaagctggagctccaccgcggtggcggcagcactagagctagtggatcccccgggctgtagaaattcgatatcaagcttatcgataccgtcgacctcgagggggggcccggtacccaattcgccctatagtgagtcgtattacgcgcgctcactggccgtcgttttacaacgtcgtgactgggaaaaccctggcgttacccaacttaatcgccttgcagcacatccccctttcgccagctggcgtaatagcgaagaggcccgcaccgatcgcccttcccaacagttgcgcagcctgaattaataa

**Ligation #2 & #3 J23100 driving LacZα expression**

ttgacggctagctcagtcctaggtacagtgctagctactagagaaagaggagaaatactagatgaccatgattacgccaagcgcgcaattaaccctcactaaagggaacaaaagctggagctccaccgcggtggcggcagcactagagctagtggatcccccgggctgtagaaattcgatatcaagcttatcgataccgtcgacctcgagggggggcccggtacccaattcgccctatagtgagtcgtattacgcgcgctcactggccgtcgttttacaacgtcgtgactgggaaaaccctggcgttacccaacttaatcgccttgcagcacatccccctttcgccagctggcgtaatagcgaagaggcccgcaccgatcgcccttcccaacagttgcgcagcctgaattaataa

**Ligation #4 lacZα with no promoter,with 3’ J23100 in promoter testing device.**

aaagaggagaaatactagatgaccatgattacgccaagcgcgcaattaaccctcactaaagggaacaaaagctggagctccaccgcggtggcggcagcactagagctagtggatcccccgggctgtagaaattcgatatcaagcttatcgataccgtcgacctcgagggggggcccggtacccaattcgccctatagtgagtcgtattacgcgcgctcactggccgtcgttttacaacgtcgtgactgggaaaaccctggcgttacccaacttaatcgccttgcagcacatccccctttcgccagctggcgtaatagcgaagaggcccgcaccgatcgcccttcccaacagttgcgcagcctgaattaataatactagagttgacggctagctcagtcctaggtacagtgctagctactagtgaaagaggagaaatactagatggcttcctccgaagacgttatcaaagagttcatgcgtttcaaagttcgtatggaaggttccgttaacggtcacgagttcgaaatcgaaggtgaaggtgaaggtcgtccgtacgaaggtacccagaccgctaaactgaaagttaccaaaggtggtccgctgccgttcgcttgggacatcctgtccccgcagttccagtacggttccaaagcttacgttaaacacccggctgacatcccggactacctgaaactgtccttcccggaaggtttcaaatgggaacgtgttatgaacttcgaagacggtggtgttgttaccgttacccaggactcctccctgcaagacggtgagttcatctacaaagttaaactgcgtggtaccaacttcccgtccgacggtccggttatgcagaaaaaaaccatgggttgggaagcttccaccgaacgtatgtacccggaagacggtgctctgaaaggtgaaatcaaaatgcgtctgaaactgaaagacggtggtcactacgacgctgaagttaaaaccacctacatggctaaaaaaccggttcagctgccgggtgcttacaaaaccgacatcaaactggacatcacctcccacaacgaagactacaccatcgttgaacagtacgaacgtgctgaaggtcgtcactccaccggtgcttaataacgctgatagtgctagtgtagatcgctactagagccaggcatcaaataaaacgaaaggctcagtcgaaagactgggcctttcgttttatctgttgtttgtcggtgaacgctctctactagagtcacactggctcaccttcgggtgggcctttctgcgtttata
